# Increased Genetic Instability in Exfoliated Oral Cells in Patients With Epidermolysis Bullosa

**DOI:** 10.1111/odi.70316

**Published:** 2026-04-16

**Authors:** Ana Carolina Sias Franco Franzosi, Rebeca Ferreira Badaró, Lucas Fernandes Leal, Rosalie Matuk Fuentes Torrelio, Willian Grassi Bautz, Leticia Nogueira da Gama‐de‐Souza

**Affiliations:** ^1^ Graduate Program in Dental Sciences Federal University of Espírito Santo Vitória Brazil; ^2^ Faculdades Integradas Espírito‐Santenses (FAESA) Avenida Jaír Etienne Dessaune Vitória Brazil; ^3^ Hospital Estadual Infantil Nossa Senhora da Glória Alameda Mari Ubirajara Vitória Brazil; ^4^ Department of Morphology Federal University of Espírito Santo Vitória Brazil

**Keywords:** biomarkers, cytology, Epidermolysis Bullosa, genotoxicity, oral mucosa

## Abstract

**Objective:**

To analyze the nuclear abnormalities of cytotoxicity—karyorrhexis (KR), karyolysis (KL), and pyknosis (PN)—and genotoxicity—micronucleus (MN) in exfoliated cells from different sites of the oral mucosa in patients with Epidermolysis Bullosa (EB) and a control group.

**Materials and Methods:**

This cross‐sectional observational study collected sociodemographic and clinical data through physical examination and a standardized questionnaire. Cells from the buccal, labial, and lingual mucosa were obtained via exfoliative cytology and evaluated for MN, KR, KL, and PN frequencies. Mann–Whitney and Kruskal–Wallis tests were applied.

**Results:**

A total of 20 EB individuals were selected. The control group (*n* = 20) was matched for sex and age with EB. Males (40%) and females (60%) demonstrated a similar distribution and individuals aged ≤ 19 years represented the majority (65%). An increase in KR and KL was detected in EB, with a greater prevalence in the buccal (*p* = 0.0002 and *p* < 0.0001) and labial mucosa (*p* = 0.0005 and *p* = 0.0240). EB patients showed a higher frequency of MN in the buccal mucosa (*p* = 0.0083).

**Conclusion:**

An increase in nuclear abnormalities at different sites of the oral mucosa demonstrated the genetic instability associated with EB. Exfoliative cytology represents a potential tool for oral biomonitoring in these patients.

## 1 Introduction

Epidermolysis Bullosa (EB) is a group of rare, genetically inherited disorders characterized by the mechanical fragility of epithelial tissues, leading to the development of mucocutaneous blisters, erosions, and ulcerations, often triggered by minimal mechanical trauma (Bardhan et al. 2020). EB is clinically and genetically highly heterogeneous, presenting phenotypes with varying degrees of severity and involving alterations in at least 20 distinct genes (Has and Fischer 2019). Owing to this heterogeneity, EB can be classified into four main types and several clinical subtypes. The primary classification is based on the skin layer where blistering occurs: simplex EB (EBS), intraepidermal blistering; junctional EB (JEB), cleavage within the lamina lucida of the basement membrane; dystrophic EB (DEB), sub‐lamina densa blistering; and Kindler EB (KEB), characterized by a mixed cleavage plane (Has et al. 2020).

Mutations in the genes involved in the pathogenesis of EB not only alter the expression of a specific structural protein within the basement membrane zone but also impact the entire extracellular matrix microenvironment. These molecular changes trigger inflammation, aberrant wound healing, and fibrosis mechanisms; promote increased and chronic wound formation; and play a role in the development of neoplasms such as cutaneous squamous cell carcinoma (cSCC), one of the leading causes of mortality in individuals with EB (Condorelli et al. 2019; De Azevedo, Roni, Torrelio, and Gama‐de‐Souza 2023).

In the oral cavity, EB can lead to vesiculobullous lesions ranging from small vesicles to large blisters. The prevalence and severity of these lesions are related to specific proteins that are absent or functionally altered in each EB type or subtype. Patients with the recessive dystrophic form (RDEB) are the most severely impacted. The buccal mucosa, followed by the tongue and lips, is the most common site of blister formation. In addition to these lesions, manifestations include ankyloglossia, the absence of lingual papillae, partial obliteration of the vestibule, microstomia secondary to the scarring process, and the presence of milia (Pratilas and Biezunski 1975; Wright et al. 1991; Serrano‐Martínez et al. 2003; Fortuna et al. 2015; De Azevedo, Roni, Dettogni, et al. 2023).

The analysis of cytological abnormalities exfoliated epithelial cells from the oral mucosa represents a valuable indicator for studying DNA damage and potential epithelial transformation. This is primarily due to the easy access to the tissue, as well as the fact that the sample provides a good quantity and quality of DNA (Borthakur et al. 2008). Cytological examination adds value to the diagnostic pathway, as it is a simple, fast, noninvasive technique that is well accepted by patients and considered a promising modality for mass screening of oral cancer (Haj‐Hosseini et al. 2024). The nuclear abnormalities commonly evaluated include pyknosis (PN), karyorrhexis (KR), and karyolysis (KL), which are markers of cytotoxicity, and micronuclei (MN), which are widely recognized as biomarkers of chromosomal damage. The presence of MN indicates genotoxic effects arising from either aneugenic (affecting chromosome number) or clastogenic (causing chromosome breaks) mechanisms (Schmid 1975).

Among the malignant complications of EB, cSCC is the most frequent and well‐documented, particularly in RDEB. In contrast, oral squamous cell carcinoma (OSCC) remains uncommon and poorly characterized. This discrepancy highlights the need to investigate early cytogenetic alterations that may precede malignant transformation in the oral cavity of EB patients. We believe that cytological analyses of the oral mucosa in EB patients represent a promising tool for monitoring and screening indicators of DNA damage. Although it is a non‐invasive technique, such analyses remain scarce in this group. Therefore, our objective was to compare the frequency of cytotoxic and genotoxicity events at different sites of the oral mucosa between EB and non‐EB individuals.

## 2 Materials and Methods

The study was designed as a cross‐sectional observational clinical study, conducted in accordance with the ethical principles of the Declaration of Helsinki and reported following the STROBE (Strengthening the Reporting of Observational Studies in Epidemiology) guidelines. It was developed in collaboration with Projeto Borboleta Azul of Faculdades Integradas Espírito‐Santenses (FAESA), a university extension project that serves as a reference center for the dental care of EB patients in Espírito Santo (ES), under the coordination of Prof. Lucas Fernandes Leal. Additionally, patients treated by the collaborating researcher Dr. Rosalie Torrelio at Hospital Estadual Infantil Nossa Senhora da Glória (HEINSG, Vitória) were also invited to participate. The project was approved by the Research Ethics Committee of CCS‐UFES under approval number 4.569.791. The analyses were performed at the Laboratory of Developmental Biology and Tumorigenesis (LBCDT) and the Multiuser Laboratory of Histotechnics (LHT) at the Health Sciences Center (CCS) of the Federal University of Espírito Santo (UFES).

### 2.1 Sample

The study included adults and children residing in ES, Brazil, who were diagnosed with EB regardless of type or subtype. Notably, the analysis of oral tissue in these patients is already part of the routine care protocol established by Projeto Borboleta Azul.

For subtype identification, EBS was diagnosed based on clinical features, while DEB and JEB were confirmed through molecular analysis. Epithelial cells were collected using buccal swabs and subjected to DNA extraction. Genetic subtyping was performed by targeted region capture using probe‐based hybridization, followed by next‐generation sequencing with Illumina technology. Bioinformatic analysis included read alignment and variant calling using the GRCh38 human reference genome and population allele frequency data from the gnomAD database. This sequencing protocol was developed and validated by Mendelics Análise Genômica, a certified clinical genomics laboratory.

The control group comprised non‐EB individuals, adults, and children, matched by sex and age to the EB sample. These individuals were selected from the Interdisciplinary Clinic at the UFES Institute of Dentistry (Vitória, ES).

The inclusion criteria were patients diagnosed with EB, regardless of type or subtype, who signed the informed consent form (ICF) and, for minors, the assent form (AF). The non‐EB group consisted of healthy individuals with no history of preexisting diseases or chronic medication use, who also signed the ICF and AF. The exclusion criteria included individuals with underlying diseases, those on continuous medication, and smokers. Sociodemographic data collected included sex, age, and heredity (the latter only for the EB sample).

### 2.2 Exfoliative Cytology

Initially, a routine stomatological clinical examination was performed, along with the identification of EB characteristics. The biological material was collected as follows: a topical anesthetic (10% lidocaine spray) was applied to the collection sites (sprayed three to five times) and then discarded after 5 s in the sink along with saliva. Sensitivity was assessed via blunt‐tipped sterile cotton forceps. The mouth was rinsed to hydrate the mucosa, and any residual particles were removed. The sites were then precisely brushed by a trained researcher (A.C.S.F.F.) A sterile endocervical brush (Kolplast) was used for each region.

The collected biological material was placed in a Falcon tube containing 1 mL of saline solution (0.9% NaCl in distilled water). For fixation, 1 mL of methanol:acetic acid solution (3:1, *v*/*v*) was added via a Pasteur pipette. The resulting solution from each collection was resuspended and distributed onto four histological slides (500 μL per slide). The slides were air‐dried at room temperature for 24 h in a sheltered environment.

For staining, the Feulgen–Fast Green technique was employed, as described by Beliën et al. (1995), with modifications, as follows: slides were immersed in 5 N HCl solution for 15 min to induce acid hydrolysis of purine and pyrimidine bases, followed by a 15‐min rinse with distilled water. The slides were then immersed in Schiff's reagent (Merck, Darmstadt, Hesse, Germany) for 90 min. After removing the Schiff's reagent, the slides were submerged in 0.01% Fast Green dye (Merck, Darmstadt, Hesse, Germany) for 30 s, followed by rinsing with distilled water. The slides were then dehydrated in four consecutive absolute alcohol baths (2 min each) and three xylene baths (5 min each). Finally, the slides were mounted with DPX (Sigma–Aldrich; Saint Louis, MO, USA).

### 2.3 Evaluation of Exfoliative Cytology

For nuclear feature analysis and image acquisition, a PrimoStar microscope (Zeiss) equipped with a Zeiss AxioCam ERC5s digital camera (Carl Zeiss Vision GmbH, Germany) and Axio Vision 4.8 Release 4.8.2 software (Carl Zeiss Vision GmbH, Germany) was used at the LBCDT of UFES.

At 400× magnification, a total of 1000 exfoliated epithelial cells were analyzed under a microscope to determine the micronucleus (MN) frequency (a genotoxicity parameter), according to the criteria of Sarto et al. (1987), and to assess nuclear abnormalities indicative of cytotoxicity—including karyorrhexis (KR), karyolysis (KL), and pyknosis (PN)—as described by Tolbert et al. (1992). The analyses were performed blindly by two examiners (A.C.S.F.F. and R.F.B.). The criteria for classifying a cell as normal in the final count were as follows: (1) intact cytoplasm with a relatively flat appearance; (2) minimal or no overlap with adjacent cells; (3) minimal or no debris; and (4) an intact, regularly contoured nucleus with a distinct perimeter (Tolbert et al. 1992).

The cytotoxicity criteria reflect different stages of nuclear degeneration and cell death. PN is characterized by increased nuclear staining intensity and reduced nuclear volume and occurs during epithelial cell differentiation and maturation, leading to cell death and exfoliation. KR is defined by nuclear fragmentation into small rounded or oval bodies within an intact cytoplasm, indicating an intermediate stage of cell death due to apoptosis or necrosis. KL represents nuclear dissolution, identified by the absence of the nucleus, indicating an advanced stage of cell death by necrosis (Tolbert et al. 1992).

To classify a cell as micronucleated, the following criteria were met: the cell exhibited normal cytoplasmic and nuclear features, and the MN had the following characteristics: (1) chromatin structure and staining intensity similar to or weaker than the main nucleus; (2) clearly defined edges, suggesting the presence of a nuclear envelope; (3) circular shape; and (4) presence in the same cytoplasm as the main nucleus, indicating that it was in the same focal plane. Additional defining criteria included lack of overlap or contact with the main nucleus, a size smaller than one‐third of the main nucleus diameter, and discernible shape and staining (Sarto et al. 1987).

### 2.4 Statistical Analysis

Data analysis and graph construction were performed via GraphPad software (California, USA). Comparisons between the EB and non‐EB groups were conducted via the nonparametric Mann–Whitney *t*‐test. Intra‐ and inter‐group comparisons were performed via the Kruskal–Wallis test followed by Dunn's post hoc test. Statistical significance was set at *p* < 0.05.

## 3 Results

In total, 20 individuals diagnosed with EB were selected. The control group (*n* = 20) was subsequently defined and matched with the EB sample (Table S1).

Sociodemographic data revealed a similar distribution of EB between females (*n* = 12, 60%) and males (*n* = 8, 40%). Individuals aged ≤ 19 years composed the majority (*n* = 13, 65%).

Regarding hereditary factors, most participants reported a family history of EB, and only one patient (5%) had parents who carried the gene but did not manifest the disease. Table 1 summarizes these findings. All nuclear aspects were identified in both EB and non‐EB samples, as illustrated in Figure 1. The quantification of each nuclear abnormality across the different anatomical sites and study groups is presented in Table 2 (Tables S2 and S3).

**TABLE 1:** Clinical and sociodemographic data (*n* = 20).

	EB	
	*N*	%
Sex		
Male	8	40
Female	12	60
Age group		
Children and adolescents (≤ 18 years)	7	35
Young adults and adults (19–59 years)	12	60
Elderly (≥ 60 years)	1	5
Type of EB		
EB simplex	13	65
Junctional EB	1	5
Dominant dystrophic EB	1	5
Recessive dystrophic EB	5	25
Educational level		
Incomplete primary	6	30
Completed secondary	4	20
Completed higher education	5	25
Incomplete higher education	4	20
Not applicable	1	5
Hereditary background		
Family history of EB	13	65
Only case of EB in family	4	20
Only case of EB, but consanguineous parents	1	5
Parents carry gene but are unaffected	1	5
Does not know	1	5

Abbreviation: EB: Epidermolysis Bullosa.

**FIGURE 1:**
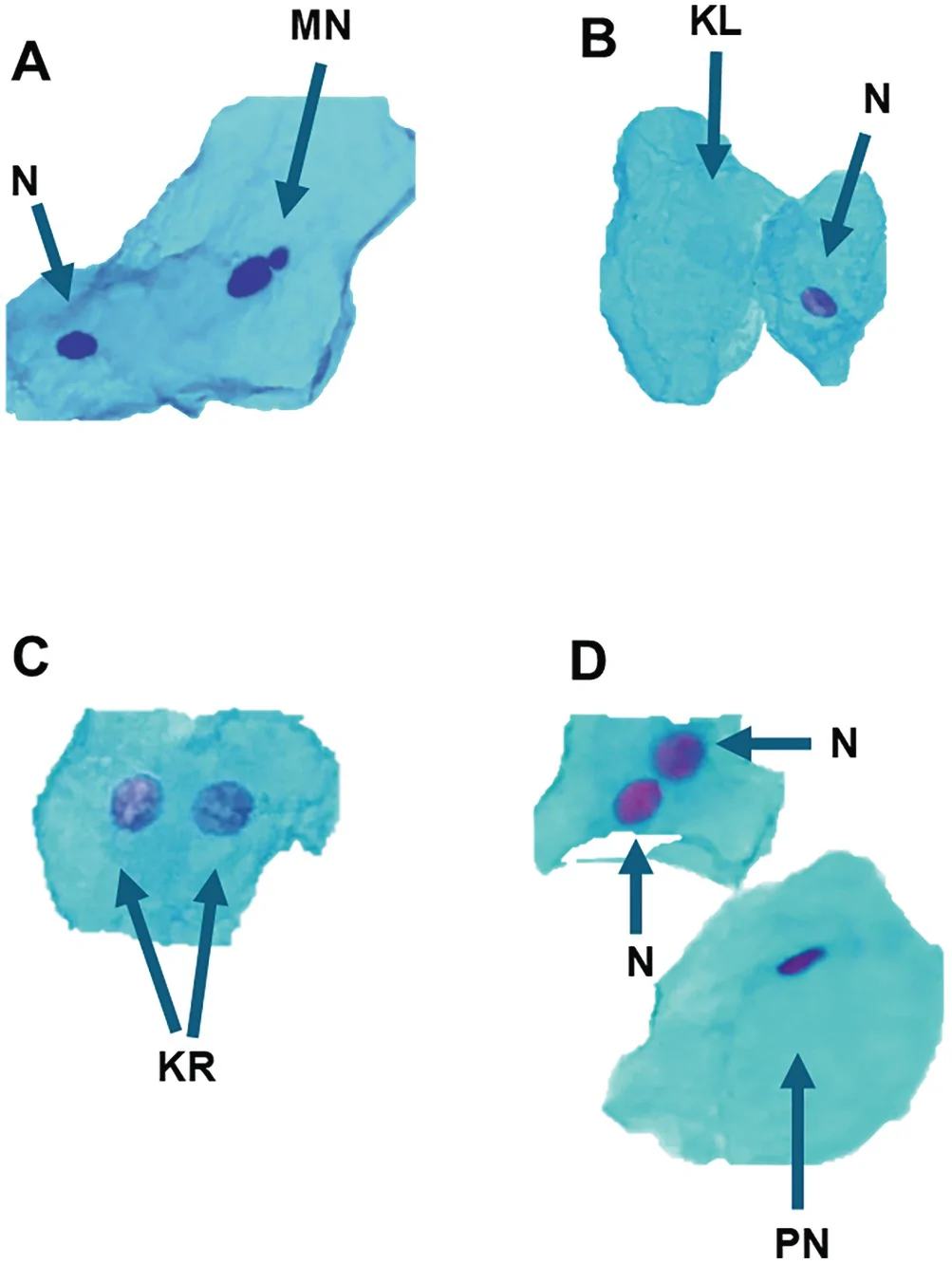
Nuclear aspects of oral mucosa smears. (A) Presence of a cell with an unchanged nucleus (N) and micronucleated cell (MN); (B) Karyolysis (KL) and normal cell (N); (C) Karyorrhexis (KR); (D) normal cells (N) and cells with pyknosis (PN). Magnification 400×. The samples were stained via the Feulgen Fast‐Green technique.

**TABLE 2:** Quantification of nuclear aspects of normality, cytotoxicity, and genotoxicity in buccal, labial, and lingual mucosa exfoliates in the EB and Non‐EB group.

Nuclear aspect	Site	EB group (mean ± SD [95% CI])	Non‐EB group (mean ± SD [95% CI])	*p*
Normal cells	BM	804.3 ± 101.9 [756.6–852.0]	917.2 ± 33.82 [901.3–933.0]	< 0.0001*
	LM	836.5 ± 55.31 [810.6–862.3]	903.0 ± 59.03 [875.4–930.6]	0.0008*
	LBT	893.5 ± 30.83 [879.00–907.9]	925.5 ± 27.3 [912.7–938.3]	0.0013*
Pyknosis	BM	23.15 ± 13.44 [16.86–29.44]	18.2 ± 13.26 [12.0–24.40]	0.2336
	LM	17.6 ± 15.94 [10.14–25.06]	18.55 ± 11.21 [13.30–23.80]	0.4406
	LBT	25.8 ± 20.3 [16.30–35.30]	26.45 ± 12.58 [20.56–32.34]	0.5157
Karyorrhexis	BM	157.4 ± 101.5 [109.8–204.9]	61.9 ± 29.03 [48.32–75.48]	0.0002*
	LM	96.05 ± 47.23 [73.95–118.2]	48.55 ± 34.03 [32.62–64.48]	0.0005*
	LBT	60.75 ± 31.58 [45.97–75.53]	33.9 ± 21.99 [23.61–44.19]	0.0066*
Karyolysis	BM	14.5 ± 13.71 [8.084–20.92]	2.5 ± 1.96 [1.583–3.417]	< 0.0001*
	LM	48.9 ± 32.47 [33.70–64.10]	29.35 ± 32.59 [14.10–44.60]	0.0240*
	LBT	19.1 ± 14.2 [12.45–25.75]	13.35 ± 11.61 [7.915–18.79]	0.1185
Micronuclei	BM	0.45 ± 0.6863 [0.1288–0.7712]	0 ± 0 [0.00–0.00]	0.0083*
	LM	1.00 ± 2.34 [−0.09496 to 2.095]	0.55 ± 1.791 [−0.2882 to 1.388]	0.3683
	LBT	0.9 ± 1.119 [0.3762–1.424]	0.8 ± 1.196 [0.24–1.36]	0.7733

Abbreviations: BM, buccal mucosa; CI, confidence interval; LBT, lateral border of the tongue; LM, labial mucosa; SD, standard deviation.

* Mann–Whitney *U* test. Significant data with *p* < 0.05.

Quantitative analysis revealed a higher frequency of cytotoxic nuclear abnormalities—KR and KL—in the EB group compared to controls in the buccal (Figure 2B. *p* = 0.0002; C. *p* < 0.0001) and labial mucosa (Figure 2F. *p* = 0.0005; G. *p* = 0.0240). In the lingual mucosa, only KR was significantly increased in EB patients (Figure 2J. *p* = 0.0066), while KL showed no difference (Figure 2K. *p* = 0.1185). PN did not differ between groups in any of the sites (*p* > 0.05 for all comparisons).

**FIGURE 2:**
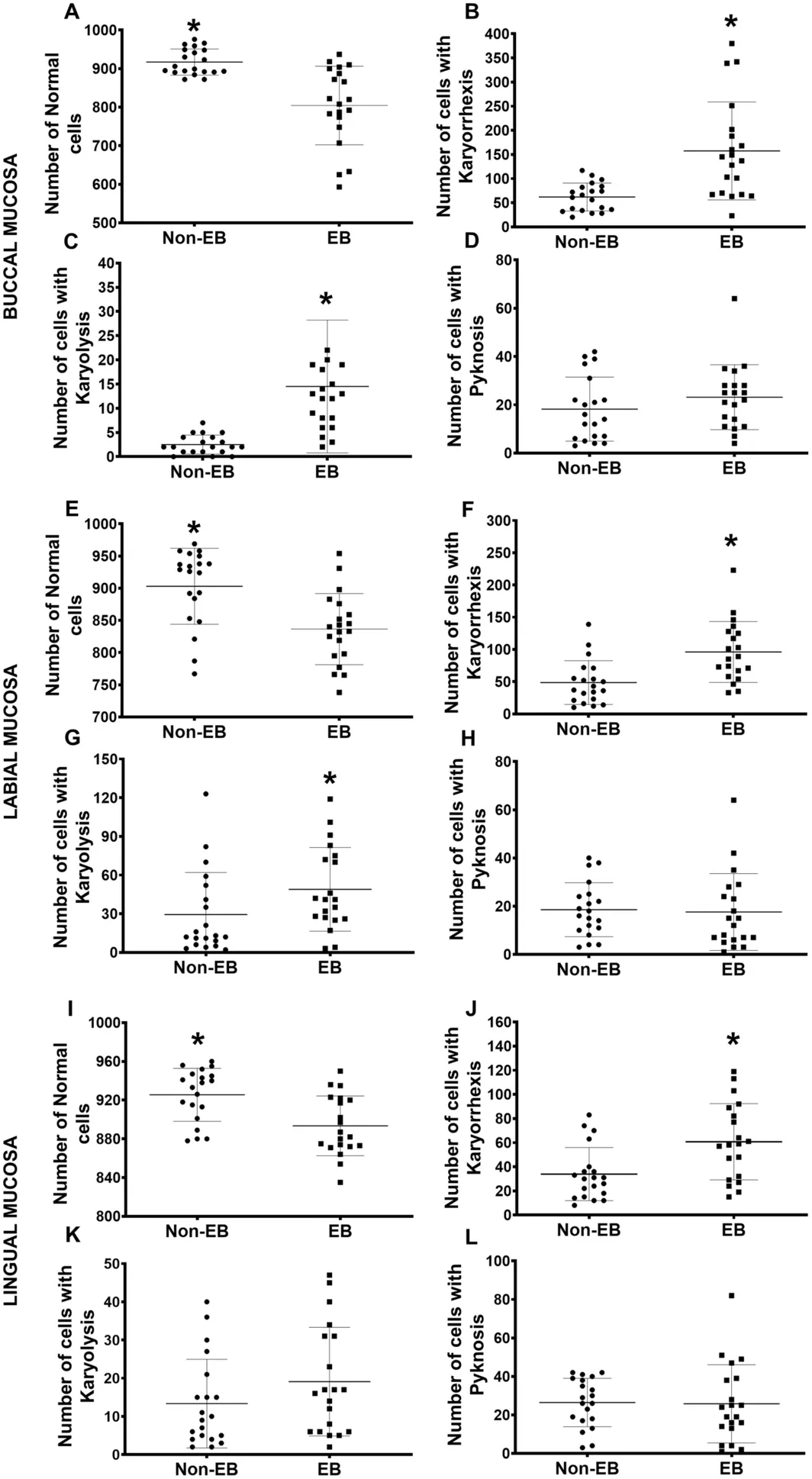
Compared with the EB samples, the non‐EB samples presented a greater number of nuclei without alterations across all the evaluated sites. The non‐EB group showed a greater number of normal nuclei in the buccal (A), labial (E), and lingual mucosa (I). The EB group showed increased KR in the buccal (B), labial (F), and lingual mucosa (J), and increased KL in the buccal (C) and labial mucosa (G). No significant differences were found for PN (D, H, L) or for KL in the lingual mucosa (K). Mann–Whitney test. *Significant data with *p* < 0.05.

MN analysis revealed a greater frequency in the buccal mucosa of the EB group than non‐EB sample (*p* = 0.0156). In contrast, no differences were found in the labial or lingual mucosa (*p* = 0.3683 and *p* = 0.7733, respectively) (Table 2).

When performing an intragroup analysis considering only cells with nuclear abnormalities, we observed that in the control sample, KR was the most frequent finding in the buccal mucosa (Figure 3A. *p* = 0.0044 and *p* < 0.0001, compared with PN and KL, respectively). The same pattern was observed in the EB group, in which KR remained predominant (Figure 3B. *p* = 0.0079, *p* < 0.0001, and *p* < 0.0001 compared with PN, KL, and MN, respectively). Additionally, PN and KL were found in greater numbers than MN (Figure 3B. *p* < 0.0001 and *p* = 0.0039).

**FIGURE 3:**
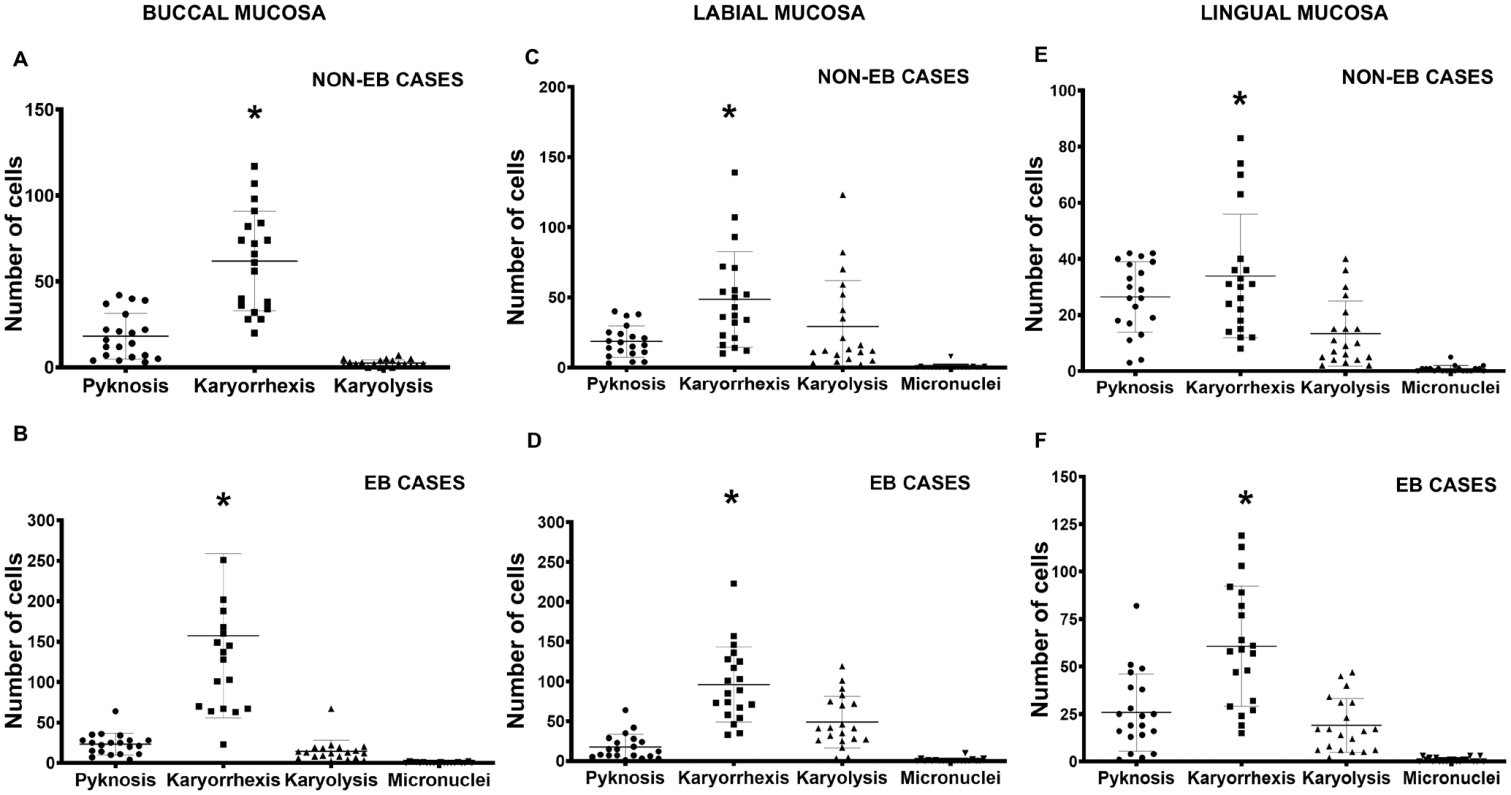
Intragroup comparison of nuclear abnormalities across different sites. KR was the most common finding in both the EB and non‐EB groups across all the evaluated sites. Kruskal–Wallis test. *Significant data with *p* < 0.05.

In the labial mucosa of the non‐EB group, cytotoxic events were more common than genotoxic events (Figure 3C. *p* < 0.0001 in all comparisons), and the EB group followed a similar pattern (Figure 3D. *p* = 0.0128, *p* < 0.0001, and *p* < 0.0001, respectively). Another finding was that KR was more common than PN (Figure 3D. *p* < 0.0001).

Regarding the lingual mucosa, in the control group, KR was more common than KL and MN (Figure 3E. *p* = 0.0418 and *p* < 0.0001), and PN and KL were detected in greater numbers than MN (Figure 3E. *p* < 0.0001 and *p* = 0.0013). The EB group presented similar findings, with KR being more prevalent than PN, KL, and MN (Figure 3F. *p* = 0.0351, *p* = 0.0050, and *p* < 0.0001, respectively), as well as PN and KL occurring more frequently than MN (Figure 3F. *p* < 0.0001 and *p* = 0.0004).

When we compared different sites within the EB sample, for KR, the buccal mucosa presented a greater number of impacted cells than the lingual mucosa (Figure 4D. *p* = 0.0002). With respect to KL, a greater frequency was detected in the labial mucosa than in the buccal mucosa (Figure 4F. *p* = 0.0002), as well as between the labial and lingual mucosa (Figure 4F. *p* = 0.0055). No significant variation was detected for PN (Figure 4H. *p* > 0.05).

**FIGURE 4:**
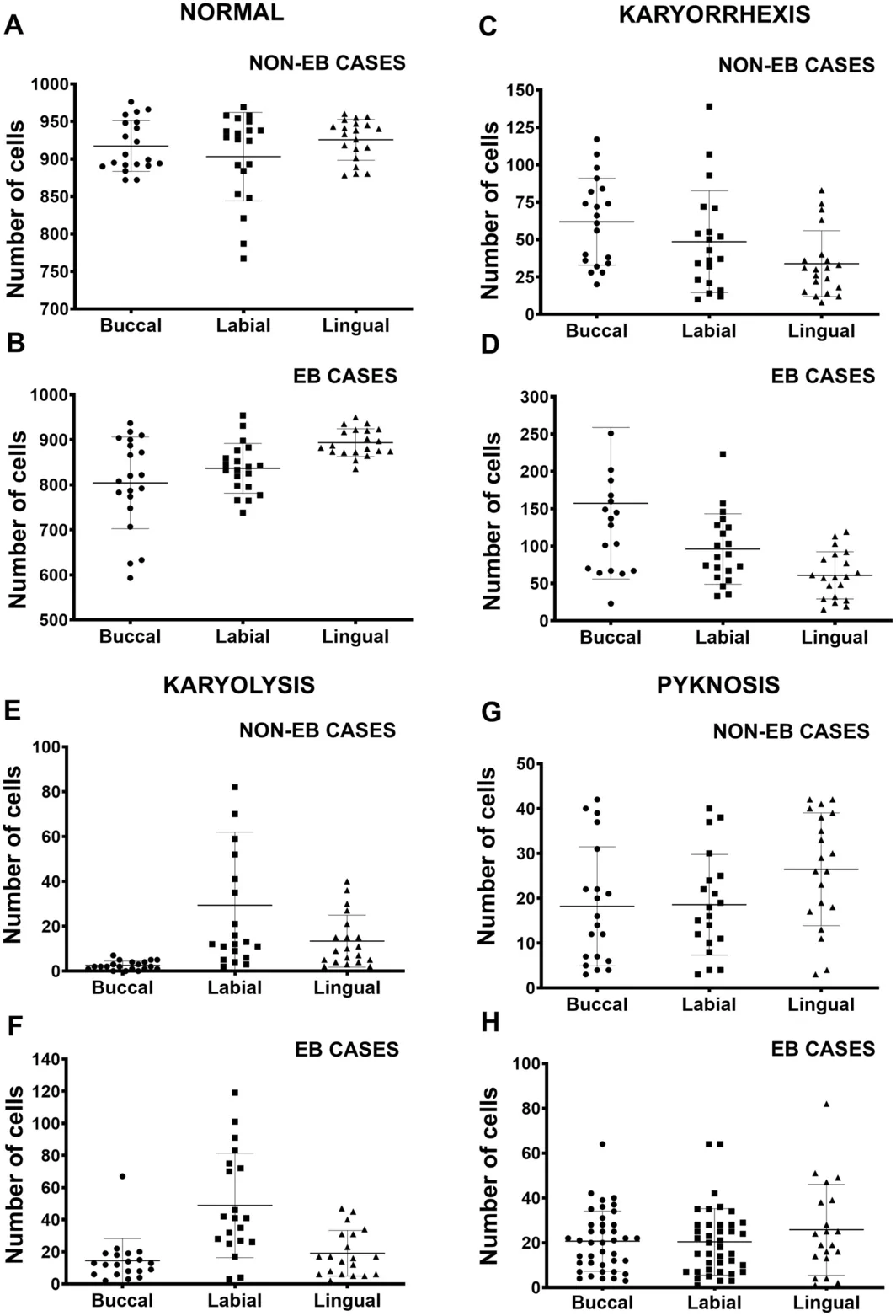
Intergroup comparison of nuclear abnormalities across different sites. In the EB group, normal cells were greater in the LM than in the BM and LBT (B, *p* = 0.0021 and *p* = 0.0064). KR frequency was higher in the BM compared to the LM (D, *p* = 0.0002), and KL frequency was greater in the LM than in the BM, as well as higher in the LM compared to the LBT (F, *p* = 0.0002 and *p* = 0.0055). In the non‐EB group, the number of normal cells was similar across sites (A, *p* > 0.9999 for all comparisons), KR was more frequent in the BM than in the LBT (C, *p* = 0.0049), and KL showed the highest frequency in the LM compared to the BM and LBT (E, *p* < 0.0001 and *p* = 0.0003). PN did not differ across sites in both groups (G and H, *p* > 0.05). BM—buccal mucosa; LBT—lateral border of the tongue; LM—labial mucosa. Kruskal–Wallis test.

In the non‐EB group, for KR, only the buccal mucosa presented a greater number of impacted cells than the lingual mucosa (Figure 4C. *p* = 0.0049). The labial mucosa presented the highest frequency of KL (Figure 4E. *p* < 0.0001 and *p* = 0.0003). And again, no differences were detected for PN (Figure 4G. *p* > 0.05).

Finally, when we compared the non‐EB group with the severe disease group (DEB + JEB), we observed a higher frequency of KL in the buccal mucosa of the EB group (Figure 5C. *p* = 0.0006). No differences were found for KR or PN (Figure 5B,D. *p* = 0.0676 and *p* = 0.134, respectively). In the labial mucosa, KR and KL were more frequently observed in the DEB + JEB group (Figure 5F,G. *p* = 0.0122 and *p* = 0.0192, respectively), whereas PN was less frequent in these patients (Figure 5H; *p* = 0.0064). No differences were found in the lingual mucosa for any of the nuclear abnormalities analyzed (Figure 5J–L. *p* > 0.05 for all comparisons).

**FIGURE 5:**
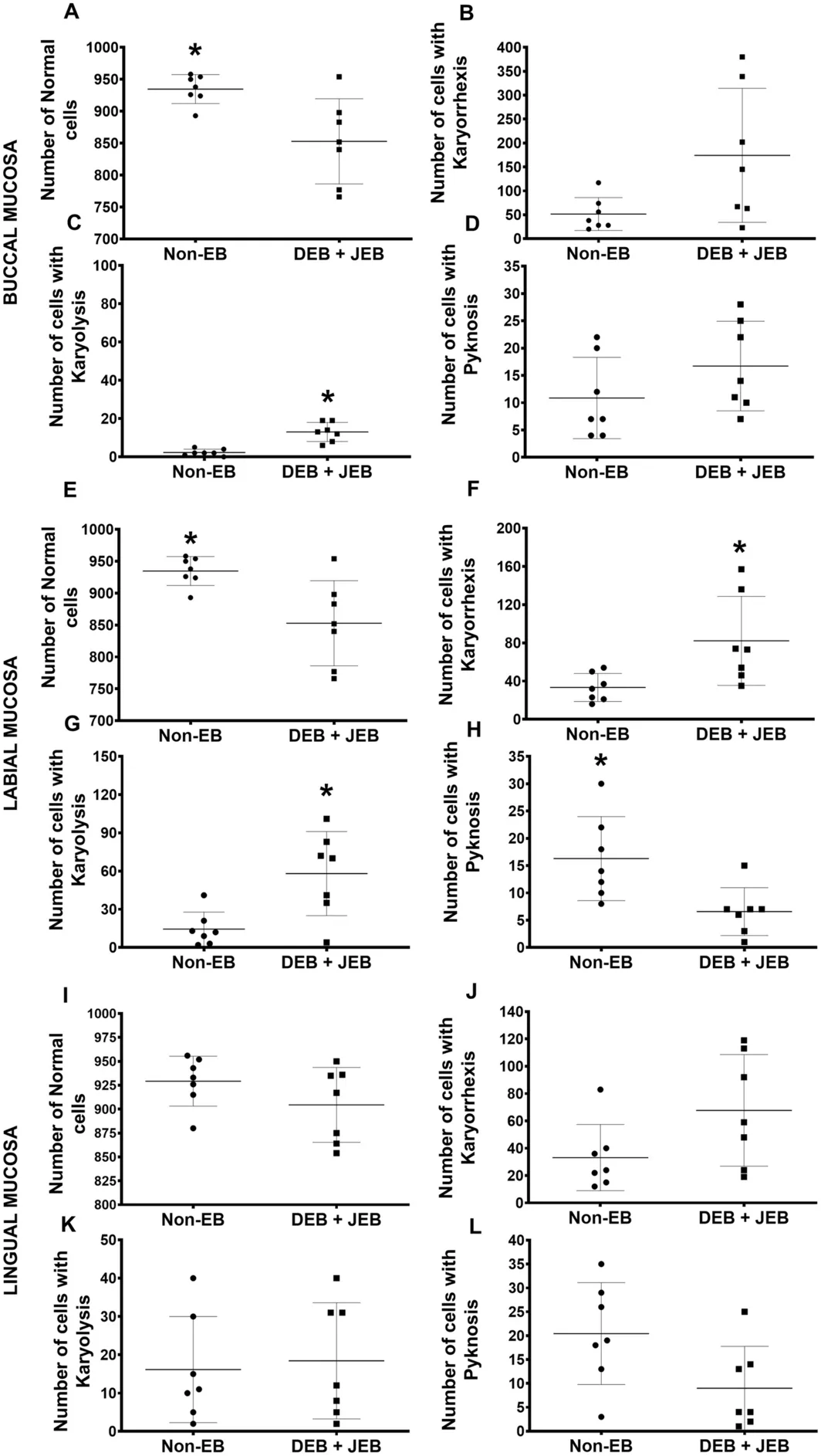
Comparative analysis between the non‐EB group and severe cases of disease (DEB + JEB) across different sites. The non‐EB group showed a greater number of normal nuclei in the buccal (A) and labial mucosa (E), and higher PN frequency in the labial mucosa (H). The EB group exhibited increased KL in the buccal (C) and labial mucosa (G), and more KR in the labial mucosa (F). No significant differences were observed for the lingual mucosa (I–L) or for KR and PN in the buccal mucosa (B, D). Mann–Whitney test. *Significant data with *p* < 0.05.

## 4 Discussion

EB is a rare and severe disease with distinct clinical characteristics, and despite recent advances in therapeutic research, it remains incurable. In the specific context of the oral mucosa, any EB subtype may lead to four typical lesions: erythema, ulcers, atrophy, and blisters. The extent and severity can range from mild to extremely severe (Wright et al. 1991; Fortuna et al. 2013; De Azevedo, Roni, Torrelio, and Gama‐de‐Souza 2023). Given the diversity of the disease and its clinical manifestations, which cause significant morbidity in patients, the search for minimally invasive biomonitoring protocols is crucial in EB management.

In this context, cytological analysis of oral exfoliated cells has emerged as a promising auxiliary tool due to its low cost and capacity to provide samples with good cell quantity and quality. Our findings reinforce these advantages, as we detected a greater frequency of markers indicating genetic instability in EB samples.

Regarding sociodemographic data, our results indicated a similar distribution of the disease between the sexes, which is consistent with the literature (Fine 2016). Although the sample was mostly composed of adults, pediatric cases were also included, which contrasts with previous studies that reported a higher prevalence in younger age groups (Uitto, Bruckner‐Tuderman, et al. 2016; Has et al. 2020). In terms of hereditary factors, most participants reported a family history of EB, aligning with the genetic nature of the disease (Bardhan et al. 2020). However, a quarter of this sample reported being the only affected individuals in their families, which may be attributed to genetic variations, in which rare cases result from spontaneous mutations in reproductive or early embryonic cells (Uitto, Vahidnezhad, and Youssefian 2016).

The wound healing process, including inflammation, proliferation, and remodeling, is significantly impaired in EB individuals at different stages. Skin and mucosal fragility, coupled with repeated blister formation, leads to chronic inflammation, delayed re‐epithelialization, and nonhealing ulcers that may become infected (Bardhan et al. 2020). In the most severe EB subtypes, such as RDEB and JEB, mutations affect genes encoding critical structural components of the dermoepidermal junction, including type VII collagen (COL7A1), laminin‐332 (LAMA3, LAMB3, LAMC2), and type XVII collagen (COL17A1). These proteins are essential for anchoring the epidermis to the underlying dermis and maintaining tissue integrity. Their dysfunction disrupts cohesion and repair mechanisms, exacerbating epithelial breakdown. As a result, skin erosions and blisters progress to chronic wounds, inflammatory conditions, and fibrosis (Cianfarani et al. 2017; Has et al. 2020; De Azevedo, Roni, Torrelio, and Gama‐de‐Souza 2023). Ultimately, debilitating and potentially fatal complications may develop, such as esophageal strictures, limb deformities, and aggressive epithelial cancers (Montaudié et al. 2016).

When markers of genetic instability were assessed, the samples of EB oral mucosa showed an increased frequency of nuclear abnormalities. Although KR and KL are primarily associated with cytotoxicity, their elevated presence, alongside other nuclear anomalies, may reflect underlying cellular stress and potential genotoxic effects. The higher prevalence of these alterations in areas such as the lips and cheeks suggests continuous cellular damage, likely due to the recurrent blister formation and tissue fragility (Feijoo et al. 2011). Although the association between KR and late‐stage apoptosis remains unclear, we hypothesize that epithelial lining cells fail to complete the tissue renewal process and ultimately undergo cell death. PN and KL, in turn, may already represent dead cells (Torres‐Bugarín et al. 2014).

The MN test was first applied for human biomonitoring via exfoliation of oral mucosa cells by Stich et al. (1982). According to Bloching et al. (2000), MN in oral mucosal cells is used to study preneoplastic impacts from samples obtained directly from affected tissues. Their presence may indicate an increased risk of malignant transformation, particularly in potentially malignant disorders such as oral leukoplakia and oral submucous fibrosis (Kranthi et al. 2024). Additionally, exfoliative cytology provides a better understanding of nuclear alterations at the single‐cell level (Palve and Tupkari 2008). Recent findings reinforce the diagnostic potential of the MN assay, especially in oral potentially malignant lesions, where it demonstrated high sensitivity (90.9%) and specificity (100%), with an overall diagnostic accuracy of 95.2% (Elnaggar et al. 2023).

Our analysis revealed an increased number of micronucleated cells in the EB group, particularly in the buccal mucosa, indicating genomic instability and genotoxicity (Proia et al. 2006; Torres‐Bugarín et al. 2014). This finding reflects the susceptibility of EB patients to genotoxic damage, which could be potentially linked to the chronic inflammation characteristic of the disease. Moreover, chronic wounds resulting from impaired healing lead to structural and functional alterations, favoring an inflammatory microenvironment. This contributes to fibrosis, which in turn exacerbates poor wound healing. Collectively, these changes may promote the development of disorders with malignant potential (Cianfarani et al. 2017; De Azevedo, Roni, Torrelio, and Gama‐de‐Souza 2023). Therefore, microtrauma and repeated cycles of epithelial regeneration might act as potentiators of DNA replication stress and inflammatory responses in EB, which would increase susceptibility to carcinogenesis in mucosal tissues considered fragile.

According to the intragroup analysis comparing different mucosal sites, KR was more frequent in the buccal mucosa of EB individuals, suggesting that repeated mechanical trauma in this region could trigger cellular damage. Additionally, KL was more prevalent in the buccal and labial mucosa, indicating site‐specific vulnerability to structural changes. Intergroup comparisons between non‐EB and EB individuals revealed a greater prevalence of KR and KL in the latter, whereas the control group presented a greater number of normal cells across all analyzed sites. These findings underscore the impact of EB on tissue homeostasis, particularly in areas prone to mechanical trauma (Bardhan et al. 2020).

Compared with the non‐EB group, which had the most severe EB subtypes (DEB + JEB), the EB group presented higher frequencies of KL and KR, reflecting the constant cellular fragility of these subtypes. Moreover, the most severe forms presented fewer normal cells. Indeed, severe lesions and impaired wound healing are hallmarks of dystrophic and junctional EB, both of which are associated with a high risk of developing SCC (Montaudié et al. 2016).

Importantly, RDEB individuals face an increased risk of cancer at a much younger age than non‐EB patients. The risk of SCC in RDEB begins in adolescence and increases over time, reaching 90% by age 55, with a metastatic mortality rate of approximately 80% within 5 years of the first tumor diagnosis (Fine et al. 2009). While most lesions develop on the skin, some cases have been reported in the oral cavity, with the tongue being the most common site, followed by the lips and hard palate (Martinez et al. 1992). Additionally, most tumors arise at chronic wound sites and exhibit aggressive and invasive behavior with high metastatic rates (Fine et al. 2009).

Oral wound healing is more efficient than in other body sites due to the rapid cell turnover of the mucosa, which accelerates repair. High vascularization promotes the recruitment of inflammatory cells, growth factors, and essential nutrients, whereas saliva maintains hydration, improves nutrient delivery, and enhances cell migration during re‐epithelialization (Brand et al. 2014). De Azevedo, Roni, Dettogni, et al. (2023) evaluated the saliva of EB patients and reported no significant differences in salivary flow compared with that of controls. However, 30% of EB individuals presented reduced stimulated salivary flow, suggesting impaired glandular secretory function.

Under homeostatic conditions, the extracellular matrix composition and structure are tightly regulated via a dynamic balance between synthesis and degradation, which is temporarily altered in processes such as growth, development, and wound healing (Taha and Naba 2019). However, in diseases such as RDEB, loss of this balance can lead to abnormal extracellular matrix deposition, excessive scarring, tissue dysfunction, and disrupted cellular signaling, potentially contributing to cancer development (Montaudié et al. 2016; Griffin et al. 2020).

Chromosomal instability is a dynamic and continuous process that may not be easily captured by high‐resolution techniques such as whole‐genome or targeted sequencing. In contrast, the MN assay is a validated, standardized, and cost‐effective method widely accepted as markers of chromosome damage, readily identifiable in cells and offering an accurate reflection of chromosomal segregation errors, making it a reliable indicator of chromosomal instability (Fenech et al. 2020). Elevated MN frequencies in healthy individuals have been associated with genomic instability and an increased lifetime cancer risk, suggesting the predictive value of this assay (Bonassi et al. 2007).

In light of these findings, our cytological results underscore the importance of periodic monitoring. Oral exfoliated cells may serve as a practical and valuable tool for the follow‐up of EB patients, particularly in severe cases. In clinical practice, this approach could enable early detection of potential cytotoxic and, more importantly, genotoxic changes in these individuals. The oral mucosa offers a unique opportunity for diagnostic evaluation and serves as an exceptional model for studying mutations, as it allows correlations to be drawn between genetic and histopathological alterations (Proia et al. 2006).

Lastly, incorporating the MN test into screening protocols could be especially advantageous due to its simplicity, non‐invasive nature, and low cost (Agarwal et al. 2014). Biomarkers are well‐recognized tools for cancer prevention and aid in identifying high‐risk patients. The earlier a potentially malignant oral disorder is diagnosed, the lower the morbidity associated with treatment and the higher the survival rates (Kiran et al. 2018; Haj‐Hosseini et al. 2024).

Given the rarity and severity of the disease, and considering that the study population was limited to a single geographic area, the EB sample comprised 20 volunteers. This limited sample size restricts the statistical power of subgroup analyses, such as comparisons by EB subtype, sex, or age. We recognize that these findings represent preliminary data and that they must be validated in larger cohorts, particularly in severe cases. In such patients, the skin and mucosa are subjected to recurring cycles of inflammation, healing, and fibrosis, which favor the early development of aggressive malignant neoplasms.

Future studies should involve larger sample sizes and implement longitudinal analyses to confirm whether cytological changes in exfoliated oral cells can be a predictive tool for malignant transformation over time. Additionally, recent progress in artificial intelligence (AI)‐based approaches for automated micronucleus detection, such as the MobileViT‐MN deep learning model (Wei et al. 2025), demonstrates that reliable automation is feasible. Incorporating such AI‐driven frameworks could overcome the limitations of manual scoring, which is time‐consuming and subject to observer variability, and ultimately enable scalable cytogenetic monitoring of EB patients in clinical and research settings.

## 5 Conclusion

The study showed an increased frequency of nuclear abnormalities related to genotoxicity and cytotoxicity in oral mucosal cells from EB patients, suggesting genetic instability that may precede malignant transformation. Thus, regular monitoring and micronucleus assays emerge as auxiliary tools in the follow‐up of severe forms of the disease, since these individuals have an increased risk for developing carcinoma.

In addition, the applicability in high‐risk populations, such as EB individuals, enhances the preventive role in cancer surveillance programs. The oral mucosa provides a unique diagnostic window, and routine cytological screening may facilitate the early identification of potentially malignant disorders and timely clinical intervention.

## Author Contributions

**Ana Carolina Sias Franco Franzosi:** writing – original draft, writing – review and editing, visualization, validation, methodology, investigation, formal analysis, data curation, conceptualization. **Rebeca Ferreira Badaró:** writing – review and editing, writing – original draft, visualization, validation, methodology, investigation, formal analysis, data curation, conceptualization. **Lucas Fernandes Leal:** writing – review and editing, methodology, supervision, conceptualization, data curation. **Rosalie Matuk Fuentes Torrelio:** writing – review and editing, supervision, methodology, conceptualization, data curation. **Willian Grassi Bautz:** writing – review and editing, methodology, supervision, conceptualization, data curation. **Leticia Nogueira da Gama‐de‐Souza:** writing – review and editing, writing – original draft, validation, visualization, supervision, resources, project administration, funding acquisition, methodology, formal analysis, data curation, conceptualization.

## Conflicts of Interest

The authors declare no conflicts of interest.

## Supporting information


**Table S1:** Detailed clinical and sociodemographic data.

**Table S2:** Quantification of nuclear aspects in the EB group.

**Table S3:** Quantification of nuclear aspects in the non‐EB group.

## Data Availability

The data that support the findings of this study are available on request from the corresponding author. The data are not publicly available due to privacy or ethical restrictions.
