# Supplementary material for: Increased Genetic Instability in Exfoliated Oral Cells in Patients With Epidermolysis Bullosa
Source: Oral Dis. 2026 Apr 16;32(8):2311–22. doi: 10.1111/odi.70316 (PMC13693628; doi:10.1111/odi.70316)
Supplement: Supplementary file 1 [file ODI-32-2311-s001.docx]

##### **Table S3.** Quantification of Each Nuclear Aspect Across All Sites in the Non-EB Group

|  | **Normal** | | | **Pyknosis** | | | **Karyorrhexis** | | | **Karyolysis** | | | **Micronuclei** | | |
| --- | --- | --- | --- | --- | --- | --- | --- | --- | --- | --- | --- | --- | --- | --- | --- |
|  | BM | LM | LBT | BM | LM | LBT | BM | LM | LBT | BM | LM | LBT | BM | LM | LBT |
| 01 | 930 | 924 | 880 | 12 | 56 | 35 | 56 | 37 | 83 | 2 | 9 | 2 | 0 | 0 | 0 |
| 02 | 976 | 938 | 933 | 4 | 20 | 3 | 20 | 23 | 24 | 0 | 21 | 40 | 0 | 0 | 0 |
| 03 | 966 | 954 | 926 | 4 | 28 | 18 | 28 | 16 | 40 | 2 | 12 | 11 | 0 | 8 | 5 |
| 04 | 872 | 950 | 952 | 7 | 117 | 26 | 117 | 32 | 12 | 4 | 3 | 10 | 0 | 1 | 0 |
| 05 | 941 | 958 | 956 | 20 | 38 | 13 | 38 | 21 | 15 | 1 | 13 | 15 | 0 | 0 | 1 |
| 06 | 872 | 937 | 960 | 42 | 82 | 26 | 82 | 14 | 8 | 4 | 11 | 4 | 0 | 0 | 2 |
| 07 | 895 | 848 | 938 | 37 | 66 | 40 | 66 | 71 | 18 | 2 | 59 | 3 | 0 | 1 | 1 |
| 08 | 963 | 893 | 915 | 7 | 28 | 19 | 28 | 54 | 36 | 2 | 41 | 30 | 0 | 0 | 0 |
| 09 | 890 | 853 | 901 | 14 | 91 | 42 | 91 | 72 | 30 | 5 | 35 | 27 | 0 | 0 | 0 |
| 10 | 949 | 892 | 941 | 16 | 34 | 41 | 34 | 55 | 14 | 1 | 16 | 4 | 0 | 0 | 0 |
| 11 | 892 | 787 | 945 | 3 | 98 | 4 | 98 | 139 | 36 | 7 | 70 | 15 | 0 | 0 | 0 |
| 12 | 948 | 767 | 913 | 12 | 40 | 33 | 40 | 107 | 33 | 0 | 123 | 21 | 0 | 0 | 0 |
| 13 | 894 | 926 | 943 | 22 | 74 | 29 | 74 | 50 | 22 | 5 | 2 | 5 | 0 | 0 | 1 |
| 14 | 891 | 938 | 878 | 22 | 84 | 42 | 84 | 36 | 70 | 3 | 11 | 9 | 0 | 0 | 1 |
| 15 | 923 | 969 | 918 | 40 | 32 | 39 | 32 | 10 | 26 | 5 | 5 | 15 | 0 | 0 | 2 |
| 16 | 899 | 958 | 955 | 39 | 61 | 30 | 61 | 12 | 12 | 1 | 6 | 2 | 0 | 0 | 1 |
| 17 | 959 | 821 | 889 | 5 | 36 | 11 | 36 | 93 | 63 | 0 | 82 | 36 | 0 | 0 | 1 |
| 18 | 884 | 934 | 947 | 6 | 107 | 17 | 107 | 43 | 31 | 3 | 4 | 5 | 0 | 0 | 0 |
| 19 | 906 | 929 | 880 | 21 | 72 | 38 | 72 | 34 | 74 | 1 | 12 | 7 | 0 | 0 | 1 |
| 20 | 893 | 884 | 940 | 31 | 74 | 23 | 74 | 52 | 31 | 2 | 52 | 6 | 0 | 1 | 0 |

BM: buccal mucosa; LM: labial mucosa; LBT: lateral border of the tongue.
