# Supplementary material for: Increased Genetic Instability in Exfoliated Oral Cells in Patients With Epidermolysis Bullosa
Source: Oral Dis. 2026 Apr 16;32(8):2311–22. doi: 10.1111/odi.70316 (PMC13693628; doi:10.1111/odi.70316)
Supplement: Supplementary file 2 [file ODI-32-2311-s002.docx]

##### **Table S1.** Detailed clinical and Sociodemographic data

| **Volunteer** | **Sex** | **EB’s Age** | **Controls Age** | **Heredity** |
| --- | --- | --- | --- | --- |
| 01 | Male | 19 years | 19 years | Family with EB |
| 02 | Female | 25 years | 24 years | Unknown |
| 03 | Male | 1 year and 8 months | 2 years | Only one with EB |
| 04 | Female | 46 years | 59 years | Only one with EB |
| 05 | Female | 21 years | 21 years | Parents with gene, no EB |
| 06 | Male | 50 years | 52 years | Family with EB |
| 07 | Female | 59 years | 59 years | Family with EB |
| 08 | Male | 11 years | 11 years | Only one with EB |
| 09 | Female | 59 years | 59 years | Family with EB |
| 10 | Male | 32 years | 33 years | Family with EB |
| 11 | Female | 57 years | 59 years | Family with EB |
| 12 | Female | 11 years | 9 years | Only one with EB |
| 13 | Female | 11 years | 10 years | Only one with EB |
| 14 | Female | 20 years | 18 years | Family with EB |
| 15 | Male | 18 years | 16 years | Family with EB |
| 16 | Female | 30 years | 31 years | Family with EB |
| 17 | Female | 54 years | 56 years | Family with EB |
| 18 | Male | 88 years | 90 years | Family with EB |
| 19 | Male | 12 years | 12 years | Family with EB |
| 20 | Female | 7 years | 7 years | Family with EB |
