# Supplementary material for: Increased Genetic Instability in Exfoliated Oral Cells in Patients With Epidermolysis Bullosa
Source: Oral Dis. 2026 Apr 16;32(8):2311–22. doi: 10.1111/odi.70316 (PMC13693628; doi:10.1111/odi.70316)
Supplement: Supplementary file 3 [file ODI-32-2311-s003.docx]

##### **Table S2.** Quantification of Each Nuclear Aspect Across All Sites in the EB Group

|  | **Normal** | | | **Pyknosis** | | | **Karyorrhexis** | | | **Karyolysis** | | | **Micronuclei** | | |
| --- | --- | --- | --- | --- | --- | --- | --- | --- | --- | --- | --- | --- | --- | --- | --- |
|  | BM | LM | LBT | BM | LM | LBT | BM | LM | LBT | BM | LM | LBT | BM | LM | LBT |
| 01 | 937 | 852 | 935 | 25 | 1 | 1 | 23 | 46 | 24 | 14 | 101 | 40 | 1 | 0 | 0 |
| 02 | 904 | 840 | 936 | 10 | 3 | 4 | 67 | 74 | 48 | 19 | 83 | 12 | 0 | 0 | 0 |
| 03 | 918 | 777 | 864 | 11 | 15 | 13 | 63 | 136 | 92 | 8 | 72 | 31 | 0 | 0 | 0 |
| 04 | 808 | 898 | 917 | 28 | 7 | 14 | 145 | 54 | 59 | 19 | 41 | 8 | 0 | 0 | 2 |
| 05 | 774 | 883 | 854 | 7 | 7 | 2 | 202 | 73 | 113 | 12 | 35 | 31 | 0 | 2 | 0 |
| 06 | 820 | 931 | 873 | 28 | 8 | 39 | 128 | 58 | 82 | 22 | 3 | 6 | 2 | 0 | 0 |
| 07 | 866 | 819 | 871 | 20 | 15 | 24 | 101 | 125 | 58 | 13 | 41 | 47 | 0 | 0 | 0 |
| 08 | 593 | 766 | 875 | 14 | 7 | 4 | 380 | 157 | 119 | 13 | 70 | 2 | 0 | 0 | 0 |
| 09 | 900 | 843 | 887 | 34 | 28 | 49 | 64 | 103 | 47 | 2 | 26 | 17 | 0 | 0 | 0 |
| 10 | 887 | 765 | 920 | 36 | 42 | 28 | 67 | 101 | 15 | 8 | 91 | 34 | 2 | 1 | 3 |
| 11 | 748 | 832 | 874 | 25 | 23 | 19 | 160 | 117 | 89 | 67 | 28 | 16 | 0 | 0 | 2 |
| 12 | 625 | 825 | 922 | 15 | 5 | 16 | 342 | 128 | 57 | 18 | 42 | 5 | 0 | 0 | 0 |
| 13 | 633 | 954 | 950 | 22 | 6 | 25 | 339 | 35 | 19 | 6 | 4 | 5 | 0 | 1 | 1 |
| 14 | 783 | 795 | 923 | 64 | 64 | 38 | 149 | 85 | 32 | 3 | 46 | 6 | 1 | 10 | 1 |
| 15 | 707 | 859 | 872 | 35 | 35 | 82 | 251 | 89 | 29 | 6 | 17 | 14 | 1 | 0 | 3 |
| 16 | 787 | 876 | 902 | 21 | 29 | 51 | 188 | 67 | 27 | 4 | 25 | 17 | 0 | 3 | 3 |
| 17 | 822 | 833 | 882 | 28 | 18 | 47 | 137 | 71 | 64 | 12 | 75 | 6 | 1 | 3 | 1 |
| 18 | 910 | 845 | 880 | 11 | 3 | 19 | 70 | 33 | 77 | 9 | 119 | 23 | 0 | 0 | 1 |
| 19 | 872 | 738 | 835 | 4 | 12 | 16 | 103 | 223 | 103 | 20 | 27 | 45 | 1 | 0 | 1 |
| 20 | 792 | 798 | 897 | 25 | 24 | 25 | 168 | 146 | 61 | 15 | 32 | 17 | 0 | 0 | 0 |

BM: buccal mucosa; LM: labial mucosa; LBT: lateral border of the tongue.
